# Cervids as Babesiae Hosts, Slovenia

**DOI:** 10.3201/eid1107.040724

**Published:** 2005-07

**Authors:** Darja Duh, Miroslav Petrovec, Andrej Bidovec, Tatjana Avsic-Zupanc

**Affiliations:** * Institute of Microbiology and Immunology, Ljubljana, Slovenia;; † Institute of Wildlife Pathology, Ljubljana, Slovenia

**Keywords:** Slovenia, EU1, Babesia divergens, cervids, molecular research

## Abstract

We describe cervids as potential reservoir hosts of *Babesia* EU1 and *B. divergens*. Both babesial parasites were found in roe deer. Sequence analysis of 18S rRNA showed 99.7% identity of roe deer *Babesia* EU1 with the human EU1 strain. *B. divergens* detected in cervids was 99.6% identical to bovine *B. divergens*.

Human babesiosis is an emerging tick-transmitted disease caused by intraerythrocytic parasites of the genus *Babesia*. A bovine parasite, *Babesia divergens* has been implicated as the most common agent of this dangerous zoonosis in Europe (1). The life cycle of *B. divergens* is determined by cattle, the vertebrate host, and by European sheep ticks, *Ixodes ricinus*. Ticks are not only the vectors of *B. divergens* but also its most important nonbovine reservoir (2). Many questions regarding parasite epidemiology and biology and the host response to infection remain to be answered. Furthermore, molecular data for *B. divergens* are scarce; only 1 DNA sequence of this parasite from humans from mainland Europe has been recently deposited (3). Recently, 2 cases of human babesiosis have been reported in Italy and Austria. The etiologic agent was identified as *Babesia* EU1, a pathogen closely related to, but clearly distinct from, *B. divergens* (4). The distinction was based on analysis of the complete babesial 18S rRNA gene, which also showed that EU1 is most closely related to *B. odocoilei*, a parasite of white-tailed deer (*Odocoileus virginianus*) in the United States (5). *I. ricinus*, the most prevalent and widely distributed tick species in Europe, has already been implicated as the vector of EU1 (4,6). Moreover, *I. ricinus* has a wide range of vertebrate hosts and readily bites humans. Rapidly and accurately identifying the reservoir of *Babesia* EU1 will enable appraisal of the full range of disease control options.

## The Study

We investigated 2 species of cervids shot by professional hunters from 1996 to 2000 in the vicinity of Ljubljana, Slovenia. DNA was extracted from spleen samples of 51 roe deer (*Capreolus capreolus*) and 30 red deer (*Cervus elaphus*), as previously described (6). Babesiae were detected in cervids by using specific nested polymerase chain reaction (PCR) that allowed discrimination between *B. divergens* and EU1. Primers were designed on the basis of alignment of complete 18S rRNA gene sequences of EU1, *B. divergens,* and *B. odocoilei*. With primers PIRO-A (7) and BABSr, a 600-bp babesial 18S rRNA gene was amplified with 5 μL of DNA and PCR Master Mix (Promega, Madison, WI, USA). One microliter of PCR product was used for nested PCR with either primer set PIRO-B/BOD and PIRO-B/BDV to detect 240 bp of 18S rRNA of EU1 and *B. divergens*, respectively. Both babesial parasites were detected in roe deer (76.5%); however, more animals were infected with *B. divergens* (54.9%) than *Babesia* EU1 (21.6%). Only 16.7% red deer were infected with *B. divergens* alone. Infection with babesial parasites did not differ significantly between sexes in either roe or red deer.

To assess DNA sequence homologies with EU1 from human and ticks, distinctive amplicons of the complete babesial 18S rRNA gene derived from cervids were cloned and sequenced. Parasite DNA from 2 roe deer that were found positive with a different set of nested primers was used in PCR with CRYPTO F and CRYPTO R (6). Amplicons were ligated into a plasmid vector (TOPO TA Cloning Kit for Sequencing, Invitrogen, Groningen, the Netherlands), and *Escherichia coli*–competent cells were transformed as instructed by the manufacturer. Plasmid DNA was purified from overnight cultures of selected colonies (Wizard Plus Minipreps DNA Purification System, Promega) and analyzed for inserts by restriction analysis with EcoR1 (Promega). Sequencing on both strands was carried out in an automated sequencer using BigDye Terminator Cycle Sequencing Ready Reaction Kit (PE Applied Biosystems, Foster City, CA, USA). Two clones were included in reactions with T3, T7, and internal primers to obtain complete gene sequence. All primers designed and used for this study are listed in the Table. Sequences were analyzed with computer programs of the Lasergene 1999 software package (DNASTAR, Madison, WI, USA) and submitted to GenBank to determine accession numbers. Homology search and alignment of the complete sequence of the babesial 18S rRNA gene from 1 roe deer showed 99.7% (5 nucleotide [nt] differences) identity with EU1 from a human patient and 99.8% (4 nt differences) identity with EU1 present in *I. ricinus* ticks from Slovenia. The complete sequence of the babesial 18S rRNA gene from another roe deer was, however, nearly identical (99.6%, 7 nt differences) to babesial parasite MO1 and *B. divergens*. Phylogenetic relationships of babesiae from roe deer and from other sources are shown the Figure. By using TREECON software (8), a phylogenetic tree was constructed with the neighbor-joining method, and topology of the tree was obtained with the K80 model. Support for the tree nodes was calculated with 1,000 bootstrap replicates.

**Table:** Nucleotide sequences and optimum annealing temperatures of the primers designed and used for nested polymerase chain reaction (PCR) and those for amplifying and sequencing the complete babesial 18S rRNA gene

Primer	Nucleotide sequence (5´→3´)	Annealing temperature (°C)
BABSr*	CTC CAA TCC CTA GTC GGC A	60
BOD*	GTT ATT GAC TCT TGT CTT TAA	53
BDV*	AAT ATT GAC TGA TGT CGA GAT	53
CRYPTO F	AAC CTG GTT GAT CCT GCC AGT AGT CAT	60 (6)
PIRO A*	AAT ACC CAA TCC TGA CAC AGG G	(7)
PIRO B*	TTA AAT ACG AAT GCC CCC AAC	(7)
PIRO C	GTT GGG GGC ATT CGT ATT TAA	60
1055 F	GGT GGT GCA TGG CCG	60
1055 R	AAC GGC CAT GCA CCA C	60
1200F	CAG GTC TGT GAT GCC	60
1200 R	GGG CAT CAC AGA CCT G	60
CRYPTO R	GAA TGA TCC TTC CGC AGG TTC ACC TAC	60 (6)

*Primers used for nested PCR.

**Figure:**
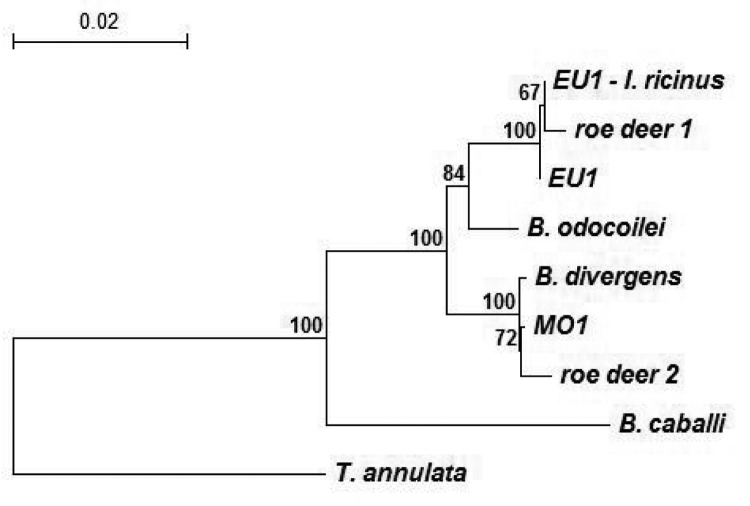
Phylogenetic relationships of representative babesiae deposited in GenBank and detected in this study, inferred from multiple sequence alignment of complete 18S rRNA gene. Accession numbers of babesiae: *Babesia* EU1 from *Ixodes ricinus* ticks, AY553915; babesiae from roe deer 1, AY572457; babesiae from roe deer 2, AY572456; *Babesia* EU1 from human, AY046575; *B. divergens*, AY046576; *B. odocoilei*, AY046577; *Babesia* MO1, AY048113; *B. caballi*, Z15104; and *Theileria annulata*, M64243. The number on each branch shows the percent occurrence in 1,000 bootstrap replicates.

## Conclusions

*Babesia* EU1, a zoonotic pathogen, was the cause of human babesiosis recently reported by Herwaldt et al. (4). While *I. ricinus* was already implicated as a vector of EU1, no other information about biology, ecology, or geographic distribution of EU1 exists (4,6). Phylogenetic analysis based on comparing the complete 18S rRNA gene sequence of EU1 derived from humans and ticks with other babesial parasites showed that EU1 is more closely related to *B. odocoilei* than *B. divergens* (4). *B. odocoilei*, which is transmitted by *I. scapularis*, primarily infects white-tailed deer in the United States (5). Cases of fatal babesiosis were described in 2 other species of cervids, namely a zoo-housed caribou (*Rangifer tarandus caribou*) and an elk (*C. elaphus elaphus*) (9). Therefore, we tested 2 species of cervids from Slovenia as potential reservoir hosts of EU1. By using specific nested PCR, the presence of EU1 was established in roe deer (21.6%) but not in red deer.

In Slovenia, roe deer are widely distributed, and the population density is high. Their pasture comprises woodland, bushes, and even open meadows and fields (10). However, red deer were nearly extinct in Slovenia in the beginning of the 19th century. Although they were later imported from Austria, Poland, and Hungary, they are still less numerous and therefore harbor fewer ticks (10). The identity of babesial parasites from roe deer from Slovenia with EU1 was confirmed by cloning and sequencing the complete babesial 18S rRNA gene. The sequences obtained were 99.8% and 99.7% identical to the 18S rRNA genes of EU1 from ticks and humans, respectively. Since the habitat of roe deer is expanding in other European countries (10), additional studies are needed to determine whether roe deer are reservoir hosts of EU1 elsewhere in Europe.

Whereas the presence of EU1 in cervids was anticipated, detection of *B. divergens* in roe and red deer was surprising. With the exception of a single report of naturally acquired babesiosis caused by *B. divergens* in reindeer (*R. tarandus tarandus*), no data about cervids as reservoirs of *B. divergens* were available at the time of our research (11,12). Although *B. divergens* can infect cervids experimentally, animals experience only mild infections with low parasitemia (2,11). However, 54.9% of roe deer and 16.7% of red deer were infected with *B. divergens* in this study. Further cloning and sequencing of the complete 18S rRNA gene of the parasite indicated 99.6% (7 nt differences) identity with babesial parasite MO1 and *B. divergens*. MO1 was described as an etiologic agent of human babesiosis acquired in Missouri and was genetically almost identical to *B. divergens* (99.9% identity, 2 nt differences), but the authors claimed that the parasites probably differ (13). However, piroplasms in abnormal hosts or hosts that are not generally considered primary hosts may have morphologic differences (12). In addition, high molecular identity of piroplasms does not necessarily mean that they have the same infectivity for different hosts.

A *Babesia* sp., tentatively called *B. capreoli*, was observed and described in red deer in Scotland (14) and sika deer (*C. nippon*) in Ireland (15). The parasite resembled *B. divergens* morphologically and antigenically. *B. capreoli* was suggested to be transmitted by *I. ricinus* ticks. The main difference between bovine *B. divergens* and these deer parasites is in their host specificity. Whereas *B. divergens* can infect a wide range of animals after splenectomy, including some deer species, various nonhuman primates, gerbils, and humans, *B. capreoli* can apparently infect only cervids and perhaps sheep (15). Nevertheless, with no deposited sequence of 18S rRNA of *B. capreoli*, the identity of *B. divergens* from roe and red deer from Slovenia is uncertain.

The finding that roe and red deer may be reservoirs for *B. divergens* has serious implications. Future research must determine if parasites from cervids share biologic characteristics with *B. divergens*, such as infectivity to cattle and humans and transmission by *I. ricinus*.

## References

[1] Kjemtrup AM, Conrad PA. Human babesiosis: an emerging tick-borne disease. Int J Parasitol. 2000;30:1323–37. 10.1016/S0020-7519(00)00137-5 11113258

[2] Zintl A, Mulcahy G, Skerrett HE, Taylor SM, Gray JS. *Babesia divergens*, a bovine blood parasite of veterinary and zoonotic importance. Clin Microbiol Rev. 2003;16:622–36. 10.1128/CMR.16.4.622-636.2003 14557289 PMC207107

[3] Centeno-Lima S, do Rosario V, Parreira R, Maia AJ, Freudenthal AM, Nijhof AM, A fatal case of human babesiosis in Portugal: molecular and phylogenetic analysis. Trop Med Int Health. 2003;8:760–4. 10.1046/j.1365-3156.2003.01074.x 12869099

[4] Herwaldt BL, Caccio S, Gherlinzoni F, Aspock H, Slemenda SB, Piccaluga P, Molecular characterization of a non–*Babesia divergens* organism causing zoonotic babesiosis in Europe. Emerg Infect Dis. 2003;9:942–8. 12967491 10.3201/eid0908.020748 PMC3020600

[5] Perry BD, Nichols DK, Cullom ES. *Babesia odocoilei* Emerson and Wright, 1970 in white-tailed deer, *Odocoileus virginianus* (Zimmermann), in Virginia. J Wildl Dis. 1985;21:149–52. 3999247 10.7589/0090-3558-21.2.149

[6] Duh D, Petrovec M, Avsic-Zupanc T. Molecular characterization of human pathogen, *Babesia* EU1, in *Ixodes ricinus* ticks from Slovenia. J Parasitol. 2005;91. In press. 10.1645/GE-394R 15986627

[7] Olmeda AS, Armstrong PM, Rosenthal BM, Valladares B, del Castillo A, de Armas F, A subtropical case of human babesiosis. Acta Trop. 1997;67:229–34. 10.1016/S0001-706X(97)00045-4 9241387

[8] Van der Peer Y, de Wachter R. TREECON for Windows: a software package for the construction and drawing of evolutionary trees for the Microsoft Windows environment. Comput Appl Biosci. 1994;10:569–70. 7828077 10.1093/bioinformatics/10.5.569

[9] Holman PJ, Swift PK, Frey RE, Bennett J, Cruz D, Wagner GG. Genotypically unique *Babesia* spp. isolated from reindeer (*Rangifer tarandus tarandus*) in the United States. Parasitol Res. 2002;88:405–11. 10.1007/s00436-001-0576-1 12049456

[10] Krystufek B. Mammals of Slovenia. 1st ed. Prirodoslovni muzej Slovenije. Ljubljana: Ministrstvo za znanost in tehnologijo; 1991.

[11] L'Hostis M, Seegers H. Tick-borne parasitic diseases in cattle: current knowledge and prospective risk analysis related to the ongoing evolution in French cattle farming systems. Vet Res. 2002;33:599–611. 10.1051/vetres:2002041 12387492

[12] Langton C, Gray JS, Waters PF, Holman PJ. Naturally acquired babesiosis in a reindeer (*Rangifer tarandus tarandus*) herd in Great Britain. Parasitol Res. 2003;89:194–8. 12541061 10.1007/s00436-002-0737-x

[13] Herwaldt B, Persing DH, Precigout EA, Goff WL, Mathiesen DA, Taylor PW, A fatal case of babesiosis in Missouri: identification of another piroplasm that infects humans. Ann Intern Med. 1996;124:643–50. 8607592 10.7326/0003-4819-124-7-199604010-00004

[14] Adam KM, Blewett DA, Brocklesby DW, Sharman GA. The isolation and characterization of a *Babesia* from red deer (*Cervus elaphus*). Parasitology. 1976;73:1–11. 10.1017/S0031182000051271 967525

[15] Gray JS, Murphy TM, Taylor SM, Blewett DA, Harrington R. Comparative morphological and cross transmission studies with bovine and deer babesias in Ireland. Prev Vet Med. 1990;9:185–93. 10.1016/0167-5877(90)90065-P

